# Expanding adverse outcome pathways towards one health models for nanosafety

**DOI:** 10.3389/ftox.2023.1176745

**Published:** 2023-08-25

**Authors:** Laura Aliisa Saarimäki, Giusy del Giudice, Dario Greco

**Affiliations:** ^1^ Finnish Hub for Development and Validation of Integrated Approaches (FHAIVE), Faculty of Medicine and Health Technology, Tampere University, Tampere, Finland; ^2^ Division of Pharmaceutical Biosciences, Faculty of Pharmacy, University of Helsinki, Helsinki, Finland; ^3^ Institute of Biotechnology, University of Helsinki, Helsinki, Finland

**Keywords:** one health, nanosafety, safe and sustainable by design, toxicoepigenomics, adverse outcome pathways

## Abstract

The ever-growing production of nano-enabled products has generated the need for dedicated risk assessment strategies that ensure safety for humans and the environment. Transdisciplinary approaches are needed to support the development of new technologies while respecting environmental limits, as also highlighted by the EU Green Deal Chemicals Strategy for Sustainability and its safe and sustainable by design (SSbD) framework. The One Health concept offers a holistic multiscale approach for the assessment of nanosafety. However, toxicology is not yet capable of explaining the interaction between chemicals and biological systems at the multiscale level and in the context of the One Health framework. Furthermore, there is a disconnect between chemical safety assessment, epidemiology, and other fields of biology that, if unified, would enable the adoption of the One Health model. The development of mechanistic toxicology and the generation of omics data has provided important biological knowledge of the response of individual biological systems to nanomaterials (NMs). On the other hand, epigenetic data have the potential to inform on interspecies mechanisms of adaptation. These data types, however, need to be linked to concepts that support their intuitive interpretation. Adverse Outcome Pathways (AOPs) represent an evolving framework to anchor existing knowledge to chemical risk assessment. In this perspective, we discuss the possibility of integrating multi-level toxicogenomics data, including toxicoepigenetic insights, into the AOP framework. We anticipate that this new direction of toxicogenomics can support the development of One Health models applicable to groups of chemicals and to multiple species in the tree of life.

## Introduction

The One Health concept is an emerging multiscale model underpinning the interconnectedness and interdependence of the health of humans, animals, and the environment. This is not only fundamental in the context of infectious diseases as exemplified by the COVID-19 pandemic, but also in chemical safety. To date, the safety assessment of chemicals and nanomaterials (NMs) has been addressed in distinct sectors focusing on specific aspects related to human health, societal, or environmental impact. However, as more and more emphasis is placed on the full life cycle assessment of NMs (Caldeira et al., 2022), the need to connect these branches becomes increasingly apparent. As a unified, transdisciplinary approach, One Health provides a crucial link for these branches and guides the development of chemicals and materials that are safe and sustainable by design (SSbD).

Traditional approaches to chemical safety assessment have been focused on the investigation of apical endpoints providing little insight to support SSbD. These traditional models have been characterized by a chemocentric view and a narrow applicability domain, resulting in a “one-chemical-one-assay”-scheme (Waters and Fostel, 2004; Sun et al., 2012). This has proven particularly problematic for NMs whose intrinsic heterogeneity has hampered the possibility to streamline their toxicological evaluation.

**FIGURE 1:**
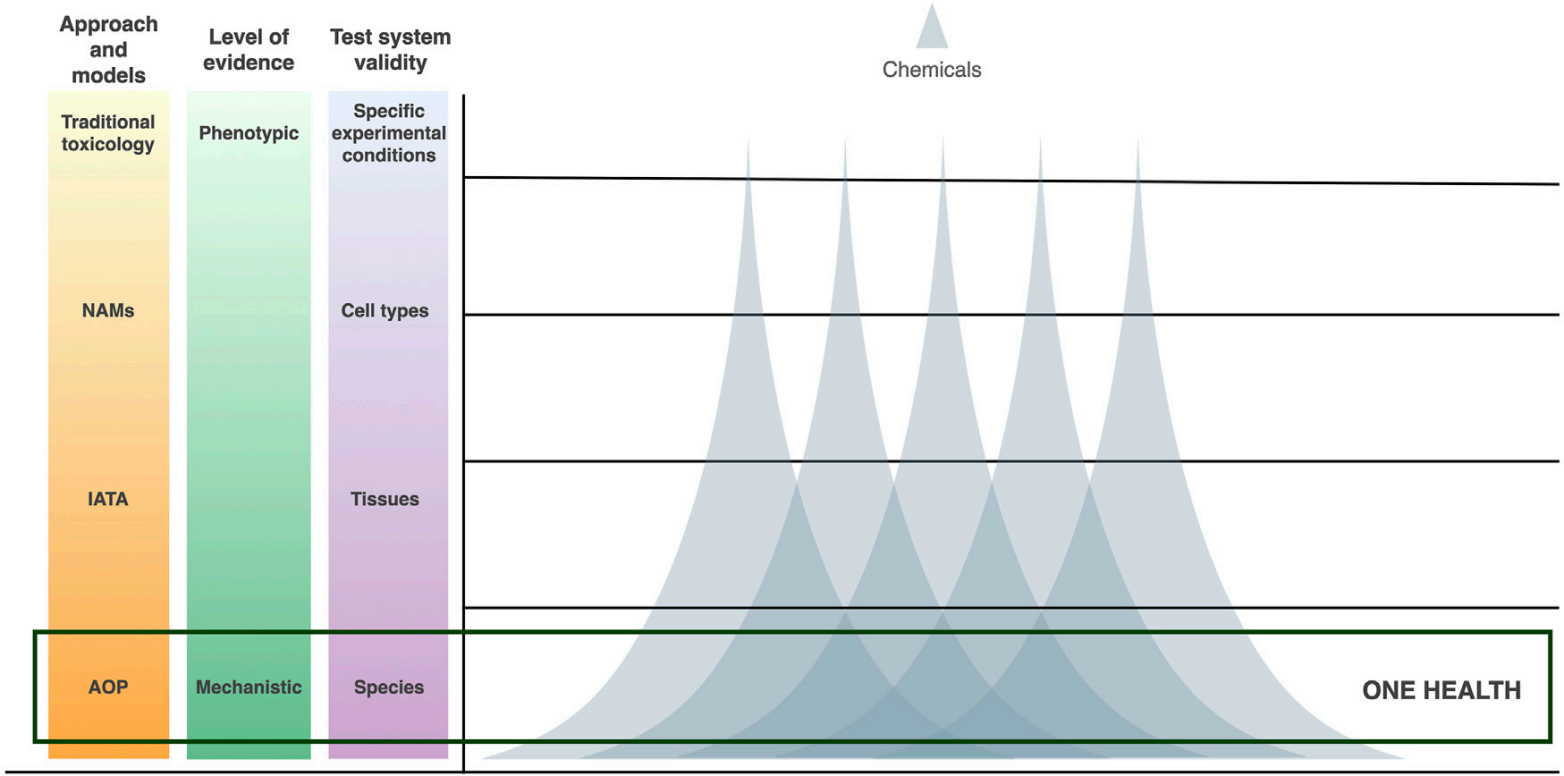
Schematic representation of adverse outcome pathways (AOPs) as One Health models for nanosafety. Expanding AOPs to accommodate increasing levels of mechanistic evidence from transcriptomic alterations to epigenetic mechanisms broadens test system validity and cross-species extrapolation. Furthermore, the reduced complexity of the possible epigenetic responses results in the identification of signatures common to a broader range of chemicals, allowing the establishment of the “many-chemicals-many-species-one-model” framework.

More recently, the introduction of mechanistic toxicology has generated increasing amounts of information on the mechanism of action (MOA) of chemicals. These molecular mechanisms can be studied by the means of toxicogenomics (TGx), a branch in toxicology that exploits omics technologies for the global investigation of biological responses. TGx provides valuable input for SSbD and supports the development of mechanistic models which have been shown to enhance *in vitro* to *in vivo* extrapolation (Kinaret et al., 2017; Di Ianni et al., 2021; Saarimäki et al., 2023). However, thus far, the focus of investigation in the field of TGx has been on more phenotypic and transient molecular layers, such as transcriptomics, proteomics, and metabolomics. While these signatures provide a comprehensive snapshot of the short-term biological responses, their complexity has led to a “one-exposure-one-signature”-approach. Omics technologies, however, intrinsically enable multiscale modelling. They can be used to address both internal (i.e., genetic and epigenetic) as well as external (environmental) factors known to contribute to pathophysiological conditions. Similarly, these molecular mechanisms can be investigated at the level of individual cells, tissues, and organs, as well as in multiple species. These concepts have inspired multi-omics approaches that expand the mechanistic investigation to the epigenetic layer, where DNA methylation, among the others, has been studied to inform on potential long-term effects and to further explain the regulation of transcriptomic changes (Scala et al., 2018; Saarimäki et al., 2020). However, to overcome the singularity of the observed signatures and migrate towards “multiple-chemicals-one-signature”, deeper investigation of the molecular machinery of the epigenome is granted. This machinery is highly conserved between biological systems (ENCODE Project Consortium, 2004; Zhou et al., 2017). Hence, instead of only a system-wide understanding provided by the more phenotypic forms of TGx, the investigation of conserved epigenetic mechanisms could bring an intra-systems level of understanding in line with the concept of One Health (Figure 1).

Besides the potential of informing on more universal mechanisms of biological response to environmental stimuli, the use of epigenetic data requires the support of more concrete, inference-based framework to link it to relevant toxicological outcomes in risk assessment. In this perspective we discuss how adverse outcome pathways (AOPs) as well-established, mechanistic multi-scale models may serve as this link, and how the introduction of toxicoepigenetics into the AOP framework could convert them into One Health models for nanosafety and beyond.

## The need for one health models in nanosafety

The rapid expansion of nanotechnology into industry, consumer goods, and medical applications has introduced nanosafety as a focus of continuous research efforts. However, as stated by Lombi *et al.* (2019), the distinct communities focused on human health and environmental safety have suffered from the lack of harmonized cross-sectorial risk governance (Lombi et al., 2019). The authors suggest that the separation of the nanosafety field into distinct communities (health and environmental nanosafety) is partly driven by different exposure scenarios (Lombi et al., 2019). Indeed, incidental exposure for humans thus far has been a small concern outside the occupational setting, while, on the opposite, exposures of ecotoxicological concern are mostly incidental. Environmental considerations in nanosafety are further challenged by uncertainties in the life cycle assessment of NMs (Nizam et al., 2021). The transformations undergone by NMs in various environments are rarely simulated under laboratory settings as these dynamic processes are difficult to reproduce (Pulido-Reyes et al., 2017).

This disconnect is apparent in the case of many of the recent nano-enabled innovations which have targeted improved human health and convenience. While the products may have been deemed generally safe, the consequences on the environment have been short-sighted. For instance, increased use of silver nanoparticles (AgNPs) as antimicrobial agents (e.g., in textiles) has potentiated their release and accumulation into the environment (Yonathan et al., 2022). The antimicrobial properties of AgNPs, however, are not unique to their intended applications. Instead, environmental release of AgNPs can harm microbial communities, affecting the nitrogen cycle and organic matter decomposition, as well as the health and function of host organisms (Yonathan et al., 2022; Hussain et al., 2023; Peng et al., 2023). Similarly, evidence of AgNPs influencing antibiotic resistance gene pools have been arising (Ma et al., 2016; Kaweeteerawat et al., 2017).

Although interactions with microbes may mediate some of the adverse effects of NMs throughout the tree of life, the potential of direct risks posed to all forms of life surge with increased production and use of NMs. Like with microbes, the disturbance of individual components of an ecosystem can induce a cascade of far-reaching events. For instance, adverse effects observed in phytoplankton and microcrustaceans may extend far into the food web and induce changes in the whole ecosystem (Corsi et al., 2022). Important considerations have been precedented by the increasing pollution caused by micro- and nanoplastics (Alimi et al., 2018). Despite differences in the lifecycle of micro- and nanoplastics in comparison to many other NMs, they serve as a warning example for the widespread consequences of uncontrolled environmental release and risk management.

The need for One Health considerations is also emerging as a central concept in the decisions of regulatory bodies. The consolidation of different aspects of nanosafety has been underpinned by the EU Green Deal Chemicals Strategy for Sustainability and its SSbD framework in accordance with the One Health concept (Caldeira et al., 2022). It highlights the need for unified approaches that promote economic growth within the environmental limits as well as social fairness and justice. It promotes complete life cycle assessment of chemicals and materials, including NMs, which necessitates modelling of complex systems at various levels of biological organization. These ambitions are accompanied with the aims of reducing and replacing animal experimentation, according to the 3R principles.

Thorough characterization of the mechanisms by which NMs exert their effects facilitates SSbD. Understanding the connection between intrinsic and biological/mechanistic properties of exposures allows to define models that can predict the behavior of chemical species designed to address specific needs. Similarly, mechanistic toxicology has opened the doors towards green chemistry and non-animal approaches, especially through the AOP framework.

## Adverse outcome pathways form the backbone of mechanistic toxicology

AOPs emerged as ecotoxicological multiscale models describing a causally linked sequence of events (key events, KEs) from a molecular initiating event (MIE) to a potential adverse outcome (AO) induced by environmental exposures (Ankley et al., 2010). They rapidly expanded to human health risk assessment and are now a central component of toxicological knowledge framework that supports mechanistic chemical risk assessment. AOPs potentiate the link from mechanistic assays to AOs according to the 3R principles by guiding the use and development of new approach methodologies (NAMs) and integrated approaches to testing and assessment (IATA). Similarly, AOPs may provide a framework for the prediction of multiple AOs with fewer tests *via* their network properties (Knapen et al., 2018). Thoroughly characterized and annotated AOP networks may hence support the evaluation of distinct processes and AOs taking place in different tissues or in multiple species upon similar (molecular) mechanisms.

AOPs serve as the interface between knowledge of biological processes and chemical safety assessment. Although they also facilitate the transition towards non-animal approaches, full characterization of some AOPs may still require extended animal assays. Similarly, the evaluation and measurement of distinct KEs often requires multiple testing strategies that need to be tailored to the specific exposure scenario. Of note, AOP-based assays and safety evaluation is only possible if the AOPs themselves are robust. However, the development and validation of AOPs is a long and laborious process often built upon extensive literature reviews. We recently demonstrated how contextualizing TGx data into the AOP framework can support the integration of TGx-derived evidence into chemical safety assessment, while also facilitating the development of new AOPs (Saarimäki et al., 2023). Molecular annotation of KEs at higher level of biological organization enables a full integration of TGx data into the framework, and further supports the interpretation of complex signatures captured by omics technologies. This TGx extension of the AOPs also brings new horizons for the translation of TGx-based insights into specific NAMs and allows the screening of multiple KEs with one exposure (Saarimäki et al., 2023).

## Toxicoepigenomics as the roadmap towards one health approaches

While TGx has traditionally focused on transient molecular districts such as proteomics, metabolomics, and transcriptomics, epigenetics is able to explain the regulatory mechanism behind them. Epigenetic regulation is also a major interface of the interconnections between species and their environment. Modifications of the epigenome induce changes in the phenotype without altering the genotype. Environmental factors, including exposures to chemicals and NMs, induce modifications of the epigenome that further influence health outcomes across life stages and possibly across generations (D’Urso and Brickner, 2014). If genetics provides a baseline that drives response to stimuli, exposures to exogenous chemicals can shape the epigenome allowing biological systems to react and adjust their gene expression programs (Loscalzo and Handy, 2014).

Response to stimuli is a multiscale process which can be assessed at different levels of granularity. However, different layers of the response (e.g., histological, cellular, molecular) show different degrees of intra- and interspecies conservation. Multiple studies have confirmed that the molecular responses observed at level of the transcriptome and proteome are often fragmented, and result in individual signatures with limited similarities (del Giudice et al., 2023; Burkard et al., 2020). However, the molecular mechanisms regulating the transcriptional responses are highly conserved (del Giudice et al., 2023; ENCODE Project Consortium, 2004).

The reduced complexity of the possible epigenetic responses implies that more generic mechanisms are put in place in response to a plethora of exogenous chemicals. Furthermore, its evolutionary conservation allows the identification of epigenetic responses shared between diverse lineages across the tree of life. (Figure 1).

We recently showed how a wide range of biological systems, spanning from humans to species of ecotoxicological interest, respond to NMs through a set of genes that are commonly regulated (del Giudice et al., 2023). In detail, we demonstrated that regardless of the variation in the transcriptomes, the response to nanoparticles is specific with respect to other compounds and is orchestrated by a family of transcription factors known as C_2_H_2_ zinc fingers. Similarly, other layers of the epigenome (microRNAs) have been hypothesized to work as interindividual and interspecies mediators of epigenetic information (Igaz and Igaz, 2015). The potential significance of miRNA for toxicogenomics has been emphasized in previous studies investigating xenobiotics induced oxidative stress, and hepatotoxicants (Hudder et al., 2008). Notably, considering their far-reaching effects on cellular physiology, it has been proposed that persistent exposure to xenobiotics could gradually modify the profile of microRNAs, resulting in an altered phenotype similar to what is observed in tumors and congenital diseases (Hudder et al., 2008). This role puts toxicoepigenomics in the heart of the One Health concept, forming the “many-chemicals-many-species-one-model” framework.

We envision that the use of epigenetic data can provide an intraspecies layer that can extend the AOP framework in a One Health prospective. Epigenetics is intrinsically multiscale, comprising marks that should be interpreted in a combinatorial way (Fessele and Wright, 2018). Since its emergence, the field of toxicoepigenetics has generated evidence for risk assessment but has lacked the ambition to inform on potential hazard alone (Lauschke et al., 2018). The humble aims have been attributed to several limitations of using toxicoepigenetic data for risk assessment, including their insufficiency (Lauschke et al., 2018). While toxicoepigenetic datasets are indeed still scarce and effort should be put to generate new data, we believe that the use of baseline epigenetic information can inform on possible mechanisms which are activated in response to NM exposures. Unlike toxicoepigenomic datasets, resources covering epigenetic states both in physiological and pathological conditions are plentiful (Grunau et al., 2001; Cancer Genome Atlas Research Network, 2008; He et al., 2008; Zhang et al., 2010; Fingerman et al., 2011; Hackenberg et al., 2011; Chadwick, 2012; Huang et al., 2015; Medvedeva et al., 2015; Zou et al., 2015). While some of these focus on specific modifications with varying levels of granularity, many databases (e.g., ENCODE) integrate functional elements in the genome, providing a valuable resource for the use and interpretation of the effect of specific epigenetic statuses (ENCODE Project Consortium, 2004). Indeed, *a priori* knowledge on gene regulatory mechanisms has been successfully integrated in pharmacology to investigate epigenetic changes in non-epigenetic drugs (Kringel et al., 2021). In toxicological settings, it was previously highlighted the inherent complexity in linking epigenetic data to the classical endpoints investigated in regulatory toxicology (Le Goff et al., 2022). Furthermore, most of the association between epigenetics and endpoints are usually in the form of correlations (Angrish et al., 2018). Finally, toxicoepigenetics often investigates low-dose and long-term effects, that do not necessarily align well with the traditional assays of regulatory toxicology (Le Goff et al., 2022). All these characteristics have hampered the application of epigenetics in risk assessment thus far.

We recently demonstrated that by linking epigenetic information into the AOP framework, we can recapitulate the most important apical endpoints of NMs exposures by a low-complexity model of molecular regulation (del Giudice et al., 2023). Hence, placing toxicoepigenetics in the context of AOPs can help to create a causative link that directly connects the chemical exposure, the epigenome, and the AO. This can support biological plausibility of epigenetic alterations while informing on possible longer term, sub-acute effects of NM exposures.

## Discussion

With increasing interest towards nanotechnology, urgent solutions to the safety assessment of NMs are needed. The safety and sustainability of emerging technologies needs to be included in the development process and assessed with a One Health outlook. Given the heterogeneous nature of NMs and their applications, transdisciplinary and generalizable models need to be established.

We believe that layers of investigation focusing on the epigenetic regulation will enable the unification of safety models towards One Health. Epigenomics can be layered with other diverse data streams and help to design a new generation of risk assessment strategies which can be applied to multiple species and multiple chemicals at once. However, toxicoepigenetics alone lacks the biological plausibility and mechanistic association to toxicologically relevant endpoints which would allow its full integration in regulatory settings.

AOPs, on the other hand, are a regulatory accepted model that bridges mechanistic information and chemical safety assessment. Furthermore, AOPs provide a flexible, evolving platform that has already shown to allow the integration of various data sources. The recent extension of AOPs into the field of infectious diseases and the introduction of modulating factors as AOP elements demonstrate their potential as a powerful framework (Nymark et al., 2021; Clerbaux et al., 2022a; Clerbaux et al., 2022b). In this light, the evidence collected here has proven that the time is right for the AOP platform to accommodate and exploit the epigenetics dimension to investigate the safety of chemicals in the long run. At the same time the increasing ambition to accommodate the 3R principles is pushing the field towards the development of new testing methods that inform on mechanistic aspects of the exposure and support SSbD. We foresee that the integration of data comprising phenotypic and mechanistic evidence, including that derived through toxicoepigenomics, will enable the multi-scale modelling of the MOA of environmental exposures. This, in turn, allows a unified representation of the chemical-biological interaction in alignment with the One Health framework.

## Data Availability

The original contributions presented in the study are included in the article/Supplementary Material, further inquiries can be directed to the corresponding author.
